# Falls during oxaliplatin-based chemotherapy for gastrointestinal malignancies – (lessons learned from) a prospective study

**DOI:** 10.1515/med-2023-0696

**Published:** 2023-05-27

**Authors:** Melanie Galliardt, Ulrich Betz, Frank Birklein, Philipp Drees, Christian Geber

**Affiliations:** Department of Neurology, University Medical Center, Johannes-Gutenberg-University, Mainz, Germany; Department of Orthopaedics and Traumatology, University Medical Center, Johannes-Gutenberg-University, Mainz, Germany; Institute of Physical Therapy, Prevention and Rehabilitation, University Medical Center, Johannes-Gutenberg-University, Mainz, Germany; DRK Schmerz-Zentrum, Auf der Steig 14-16, Mainz, 55131, Germany

**Keywords:** chemotherapy, fall-risk-index, outpatient setting, polyneuropathy, neurotoxicity

## Abstract

This prospective cohort study aimed to characterise the impact of oxaliplatin-based chemotherapy and its neurotoxic side effects (i.e., chemotherapy-induced neuropathy) on functional fall-risk and falls. Twenty chemotherapy-naïve participants (mean age, 59 years; 16 males) were consecutively included. A multimodal fall risk assessment was performed at four time points within 6 months. Polyneuropathy was assessed using the Neurologic Disability Scale; the fall risk was assessed by functional tests (Tinetti Test, Chair-Rising Test, and Timed up and Go Test). Patient-reported outcomes comprised the Hospitality Anxiety and Depression Scale (HADS), the Falls Efficacy Scale – International (FES-I) to assess the fear of falling, and the Physical Activity for the Elderly (PASE) questionnaire. Three falls occurred during the study. All fallen participants had a high fall risk-index (≥4 more risk factors) compared to only 30% of the non-fallen participants (*p* = 0.03) and suffered more frequently from pre-existing mild polyneuropathy (*p* = 0.049). Study discontinuation (*n* = 12) was associated with a higher rate of polypharmacy (*p* = 0.045), anxiety (HADS-A, *p* = 0.03), and specific fear of falling (FES-I, *p* = 0.025). In contrast, study completers (*n* = 8) reported an improvement in physical activity (PASE) (*p* = 0.018). In summary, pre-existing fall-risk factors impacted more falls than chemotherapy. A fall risk index offers a time-efficient screening option in an outpatient oncological setting.

## 1 Introduction

Falls and associated mobility impairment are a major public health concern, with the fall risk being strongly associated with age and chronic disease [1,2]. Cancer patients are at particular risk of falls, as is the general elderly population, due to the disease- and treatment-related effects on physical function [3,4,5,6]. As the population ages and cancer diagnosis and treatment strategies improve, the number of cancer survivors at a high risk of falls is increasing [5,7,8]. Non-modifiable (age and gender) and modifiable risk factors (environmental hazards, sensory deficits, balance/gait disorders, and medication problems) contribute to the high risk of falls [9]. This means that fall risk must be defined as a dynamic process and reassessed during chemotherapy.

From a neurological perspective, chemotherapy-induced neuropathy (CIPN) is one of the most common side effects in cancer treatment. The prevalence ranges from 30 to 75% [10,11] depending on chemotherapeutic agents, cumulative dose, and pre-existing medical conditions [12,13,14]. The risk increases with higher doses of neurotoxic substances, and the resulting neuropathic symptoms (CIPN) indicate an increased risk of falling at different stages of cancer treatment [15,16,17].

While the incidence, clinical symptoms, and pathomechanisms of CIPN have been intensively studied [18,19,20], the functional and dynamic changes in balance and mobility disorders associated with CIPN have been less well studied, mostly in cross-sectional and retrospective studies [21,22]. Prospective clinical observations are rare and do not functionally examine the effects of CIPN on physical performance and fall risk [8,16].

Therefore, our study aims to provide new insights into the dynamics of functional risk and risk of falls during early oxaliplatin therapy intervals using validated tests of physical performance and mobility (e.g., Tinetti test and timed-up-and-go test), self-assessment of activities of daily living (ADL), and psychometric questionnaires under close monitoring of sensorimotor impairment. The chemotherapeutic agent under investigation – the platinum derivative oxaliplatin – has a known neurotoxic risk profile, with a high CIPN incidence of up to 75% in early phases of treatment and a minimum dose-related threshold of 540 mg/m² [23]. We hypothesised that functional fall risk would increase with higher doses of chemotherapy and that the severity of CIPN would correlate with increased fall risk.

## 2 Methods

### 2.1 Study design

**Figure 1:**
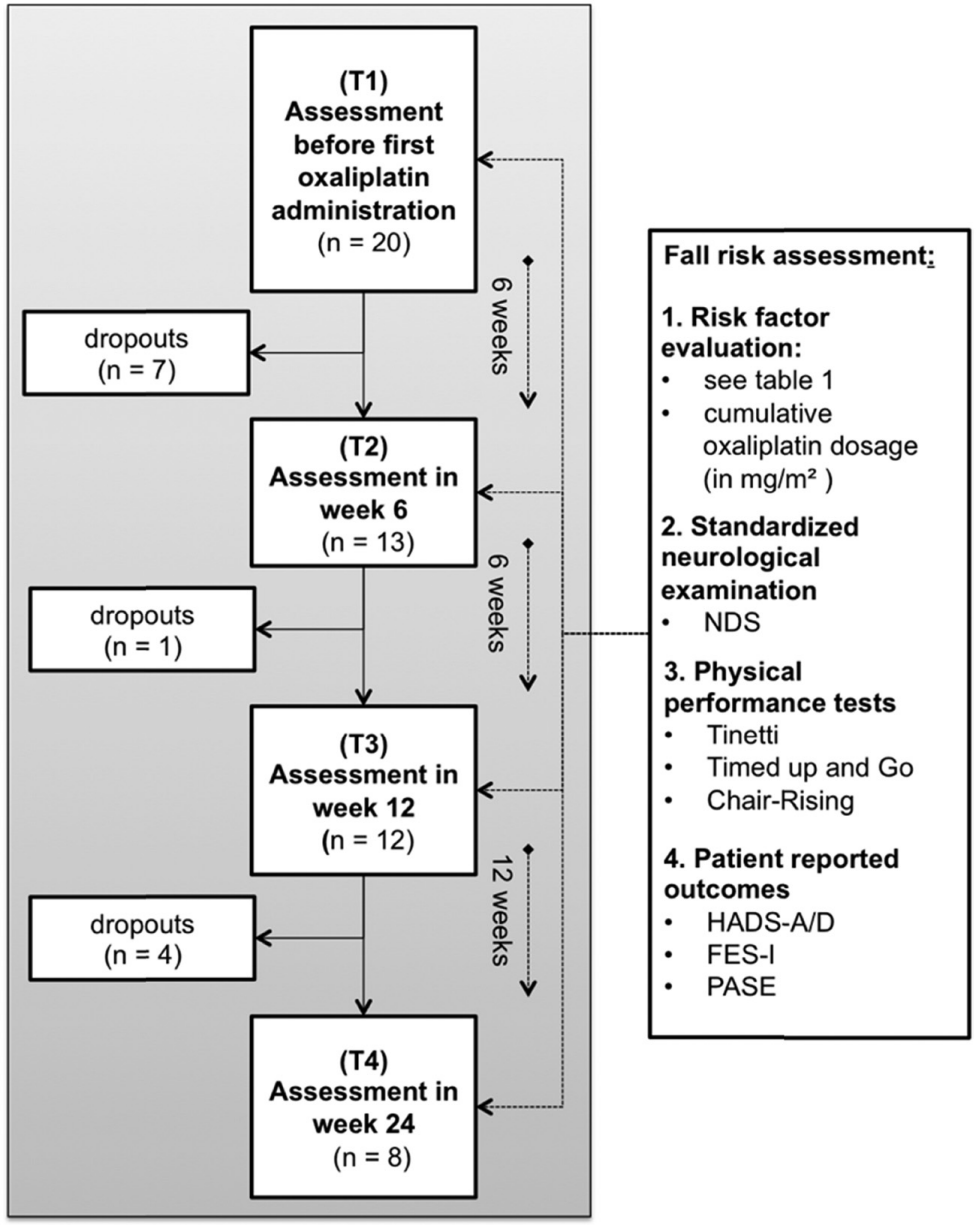
Flow chart: study design and risk assessment.

The multimodal fall risk assessment was performed at week 0 (T1) of the first chemotherapy administration and repeated at 6 (T2), 12 (T3), and 24 (T4) weeks. The study visits T2–T4 were performed with a time interval of up to 7 days from the last oxaliplatin administration to minimise bias due to acute cytostatic side effects (nausea, etc.). Each study appointment included a standardised neurological and psychometric assessment, physical performance tests (see below), and a history collection on the extrinsic and intrinsic fall risk profile of the participants. Oxaliplatin doses were recorded cumulatively (mg/m^2^) for the three follow-up dates (T2–T4) on an individual basis. Participants’ records were also reviewed for medications and concomitant circumstances (e.g., interruptions in chemotherapy). Each examination session lasted approximately 45–60 min (Figure 1).

### 2.2 Clinical assessment and performance tests

#### 2.2.1 Standardised neurological examination

The Neuropathy Deficit Score (NDS) [24] was used as a standardised neurological examination. It includes the ankle reflex, vibration threshold, temperature (cold object), and pin-prick sensation of the great toe of each leg. The reflexes were scored as present (= 2), present with reinforcement (= 1), or absent (= 0). The sensory modalities were scored as either present (= 0) or reduced/absent (= 1) on each side, resulting in a clinical score of 0–10 points, indicating mild (3–5 points), moderate (6–8 points), or severe (9–10 points) neuropathic signs. The cut-off of 3 points was set for relevant neuropathic deficits indicating polyneuropathy.

#### 2.2.2 Assessment of mobility and physical performance

The Tinetti assessment tool, also called POMA (Performance Orientated Mobility Assessment), measures an older adult’s gait and balance abilities. A low-performance score is associated with a high fall risk in an individual. The Tinetti test has been validated as a robust fall risk instrument and predictor of falls in the elderly (>65 years) [25]. The maximum score is 28, with a cut-off below 21 indicating an increased fall risk.

The additional physical performance measures and questionnaires were used to obtain a comprehensive overview of the participants’ functional risk status. The Chair Rising Test assesses the muscle strength of the lower limb by measuring the time duration of the transfer from a seated to a standing position and back to sitting five times. A cut-off of 10 s or more correlates significantly with an increased frequency of falls [26].

The Timed-up-and-Go tool (TUG) assesses dynamic balance and functional mobility. Subjects were instructed to sit back in a standard armchair and to stand up, walk a marked path of 3 m, turn around, and sit back down in the chair. The cut-off value was set at 12 s based on the reference value for geriatric adults [27].

### 2.3 Patient-reported outcomes

#### 2.3.1 Hospital anxiety and depression scale (HADS)

Patients were asked to fill in the HADS questionnaire to identify signs of common psychological burden. This psychometric tool contains 14 questions, which led to an anxiety subscale (HADS-A) and a depression subscale (HADS-D) score ranging 0–21 points each. A minimum of 7 points indicated mild depression or anxiety with higher scores indicating severe affective symptoms [28].

#### 2.3.2 Falls Efficacy Scale - International (FES-I)

The FES-I is a self-completion questionnaire that measures participants’ fear of falling in all everyday situations or activities. Sixteen items are scored on a 4-point ordinal scale (0 = no fear of falling to 4 = severe fear of falling), resulting in a maximum score (highest fear) of 64 [29].

#### 2.3.3 Physical activity scale for the elderly (PASE)

The PASE instrument assesses a person’s physical activity over the past week using a detailed quantitative and qualitative scoring system. Information on frequency, duration, and intensity of typical leisure, household, or work-related activities led to a scale ranging 0–793 points, with higher scores indicating greater physical activity [30].

### 2.4 Study population

Twenty participants (mean age, 59 years; 16 males) from the outpatient clinic for tumours of the gastrointestinal tract, pancreas, and bile ducts (University Medical Centre of the Johannes Gutenberg-University Mainz) and a medical practice for haematology and oncology at Mainz were prospectively included during a 2.5-year period (2012–2014). Participants were examined at the outpatient clinic of the Department of Neurology and the Institute for Physical Therapy, Prevention and Rehabilitation of the Johannes Gutenberg-University Medical Centre, Mainz.

Written informed consent was obtained from all participants, and the Rhineland-Palatinate Ethics Committee approved the study (processing number: 837.193.12(8294-F)).

All participants were chemotherapy-naïve and scheduled to receive a FOLFOX (FOLFOXIRI, XELOX [capecitabine and oxaliplatin]) regimen as their first-line chemotherapy. Individual oxaliplatin dosing per cycle varied 70–130 mg/m^2^.

Risk assessment at baseline included sex, age >65 years, BMI >30 kg/m^2^, history of falls, walking disability (use of a walking aid), and visual impairment (assessed with visual acuity cards). Participants’ records were reviewed for comorbidities and fall-related medication (e.g., benzodiazepines, diuretics, antiarrhythmics, and neuroleptics) and polypharmacy (>4 medications/day). Other comorbidities such as arterial hypertension, diabetes mellitus, musculoskeletal disorders, or postsurgical pain were grouped as fall risk factors because they may indicate increased frailty (Tables 1 and 2). A high fall-risk-index is defined by four or more risk factors per person [31].

**Table 1:** Risk factor profile at baseline (T1)

Risk factor	Baseline cohort (T1) (*n* = 20) No (%)
Age >65 years	*n* = 7 (35%)
Sex (female)	*n* = 4 (20%)
Obesity (BMI >30 kg/m²)	*n* = 3 (15%)
Walking aid	*n* = 3 (15%)
Visus deficit	*n* = 15 (75%)
Polypharmacy (>4 med.)	*n* = 5 (25%)
Fall-associated medication	*n* = 11 (55%)
Comorbidities^+^	*n* = 17 (75%)

^+^comorbidities such as arterial hypertension, diabetes mellitus, musculoskeletal disorders, or postsurgical pain were grouped together as fall risk factors because they may indicate increased frailty.

**Table 2:** Risk factor differences between dropouts and completers at T1

Risk factor	Completers (*n* = 8)	Dropouts (*n* = 12)	*p*-value (completers vs dropouts)
Age (>65 years)	2 (25%)	5 (42%)	0.64
Sex (female)	1 (12.5%)	3 (25%)	0.62
Obesity (BMI >30 kg/m²)	0	3 (25%)	0.24
Visual impairment	7 (87.5%)	8 (75%)	0.10
Polypharmacy (>4 med.)	0	5 (42%)	0.045*
Fall-associated medication	4 (50%)	7 (58%)	1
Walking aid	0	3 (25%)	0.24
Comorbidities	6 (75%)	11 (95%)	0.34

*p < 0.05.

Exclusion criteria comprised previous antineoplastic chemotherapy or pre-diagnosed peripheral polyneuropathy. Due to the physically challenging examination procedure, patients with acute pain, dizziness, and cognitive impairment were excluded.

### 2.5 Statistical analysis

Statistical analyses were performed using IBM SPSS statistical package version 25. Correlation analyses were applied to study the association between 1) cumulative chemotherapy doses (= Δ mg/m^2^) and changes in functional fall risk (Δ Tinetti score) and 2) changes in the polyneuropathy score (= Δ NDS score) and changes in functional fall risk (Δ Tinetti score).

Correlation analyses were performed based on the data of the follow-up cohort (*n* = 12) after 12 weeks (T1–T3). Univariate linear regression was performed to analyse the effect of cumulative oxaliplatin dosage on functional fall risk and the effect of changes in polyneuropathy severity on functional fall risk. Linear dependence of the variables was determined by Pearson correlation. Statistical significance for the two main questions was assumed at a *p*-value of <0.025 (Bonferroni correction for multiple testing).

In a retrospective explorative approach, we compared baseline data from study participants who discontinued the study (*n* = 12) with those who completed the study (*n* = 8), as well as those who fell during the study (*n* = 3) and those who did not (*n* = 17). At T1, we examined these samples for differences as possible predictors of study discontinuation or falls. Fisher’s exact test (categorical variables) or the Mann–Whitney U-test (continuous variables) was used.

We also performed a follow-up assessment of changes in risk factors after 12 weeks (*n* = 12) and in the sample of completers (*n* = 8) during 24 weeks of oxaliplatin-based chemotherapy. The McNemar (categorical variables) and Wilcoxon signed-rank (continuous variables) tests were applied. Without adjustment for multiple testing, a *p*-value of <0.05 was considered significant.

**Figure 2:**
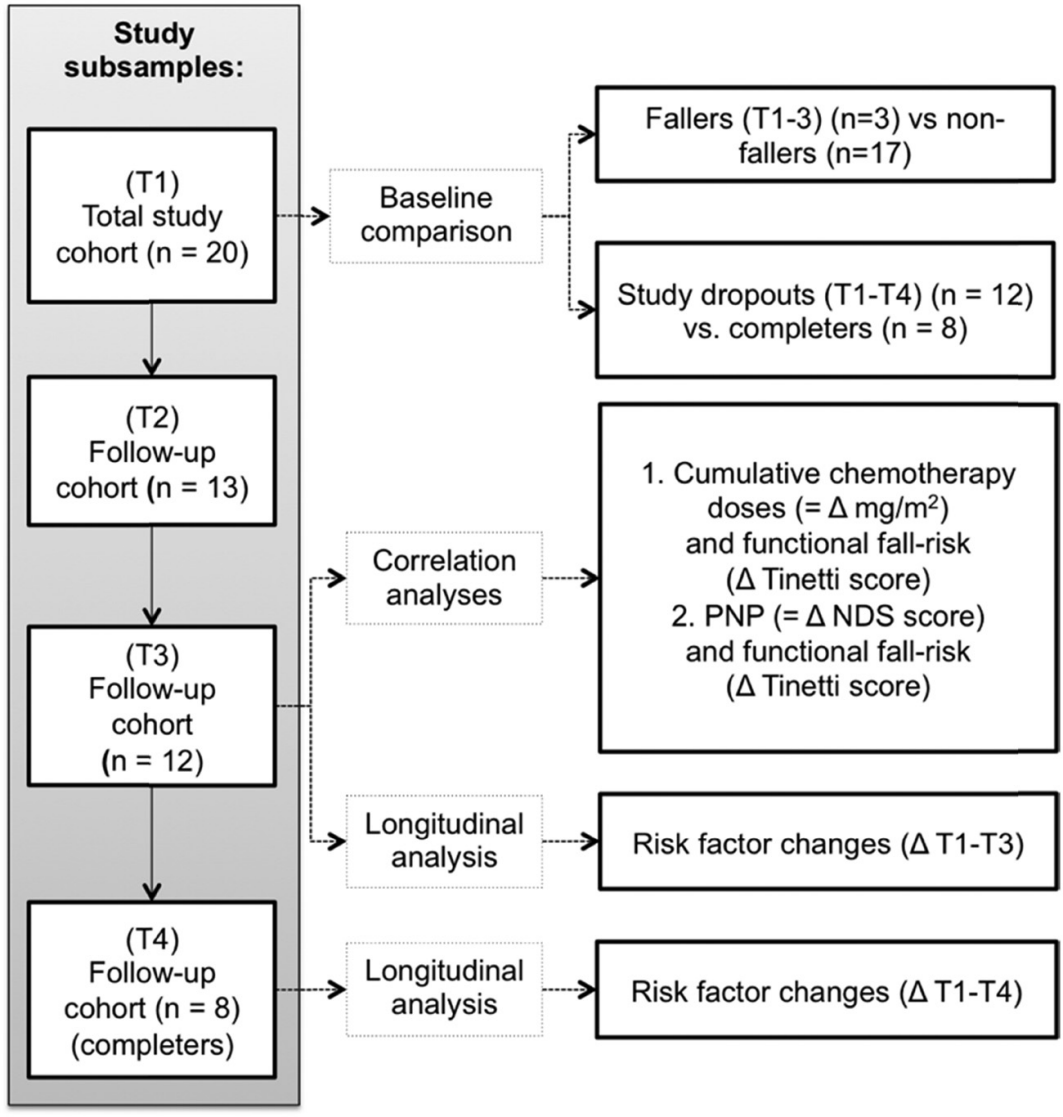
Consort chart of sub-samples and analyses.

Data are presented using means and standard deviations (SD) for continuous data and percentages for dichotomous data, e.g., cut-off values (Figure 2).

## 3 Results

In total, 60% (*n* = 12) of all participants dropped out of the study. Reasons for discontinuation included worsening health (*n* = 5), death (*n* = 2), change of chemotherapeutic agent (*n* = 1), and at their own request/no information available (*n* = 4), with eight participants discontinuing in the first half of the study (before T3).

At baseline, seven participants (35%) presented with a high fall-risk index (T1), defined by four or more risk factors per person [31].

The two main correlation analyses revealed no association between 1) cumulative chemotherapy dosages and changes in functional fall risk (*r* = 0.51; *p* = 0.088) and 2) changes in polyneuropathy severity and functional fall risk (*r* = 0.23, *p* = 0.47).

### 3.1 Explorative analyses

#### 3.1.1 Baseline comparison of study dropouts vs completers (T1)

Participants who discontinued the study (*n* = 12) initially reported higher psychological distress, such as anxiety (HADS-A: 7.5 ± 4.2 vs 3.63 ± 4.3; *p* = 0.031) and fear of falling (FES-I: 21.8 ± 4.6 vs 16.8 ± 4.7; *p* = 0.025). Polypharmacy was more frequent in participants who discontinued (*n* = 5; 42%) than among those who did not (*n* = 0; *p* = 0.045) (Tables 2 and 3).

**Table 3:** Risk assessment differences between dropouts and completers at T1

Fall-risk-assessment (T1, *n* = 20)	Completers (*n* = 8)	Dropouts (*n* = 12)	*p*-value (completers vs dropouts)
Tinetti score	25.75 ± 3p	21.55 ± 7p	0.21
Neuropathy deficit score (NDS)	1.25 ± 1.5p	3.67 ± 3.6p	0.14
HADS-A	3.63 ± 4.53p	7.5 ± 3.2p	0.03*
HADS-D	4.5 ± 4.63p	6.17 ± 3.9p	0.34
FESI (range 16–64p)	16.75 ± 1.17p	21.75 ± 5.05p	0.025*
PASE (range 0–793p)	107 ± 55p	70.3 ± 49p	0.152
Chair-rising test (CRT)	11.88 ± 1.9s	14.25 ± 4s	0.14
Timed up and Go (TUG)	9.1 ± 3.3s	11.75 ± 6.3s	0.38

p: points; s: seconds.

*p < 0.05.

#### 3.1.2 Baseline comparison of fallers versus non-fallers

A fall was defined as an event that results in a person unintentionally coming to rest on the floor, ground, or other lower level. We excluded falls caused by serious intrinsic events such as epileptic seizures, strokes, or paralysis and by precipitating external events such as car accidents, sports, or weather conditions. Accordingly, three falls were reported in the first half of the study (T1–T3) and were eligible for inclusion. Two further falls were not eligible for analysis because they were clearly triggered by environment (black ice) and behaviour (tennis).

A retrospective comparison of the cohort’s (*n* = 20) fall risk profile at baseline (T1) revealed differences between participants who fell and those who did not. Falls were associated with a high fall risk index (i.e., ≥4RF/person) in fallen (100%) versus non-fallen participants (30%; *p* = 0.003) and mild pre-existing PNP clinically defined by an NDS score of 3–5/10 points (100% vs 42%; *p* = 0.049).

#### 3.1.3 Longitudinal analysis of risk factor changes over 12 weeks (*n* = 12)

None of the assessed risk factors (ΔT1–T3 [Table 2]) changed during the 12-week observation period (mean oxaliplatin administration 563 ± 128 mg/m^2^).

The results of the neurological examination did not reveal a clinically measurable CIPN development (Table 4).

**Table 4:** Fall-risk profile changes in the T3-follow-up cohort (T1–3)

Fall-risk-assessment (*n* = 12)	T3	ΔT1–T3	*p*-value (ΔT1–T3)
Tinetti score	25.5 ± 4.2p	0.4 ± 0.7p	0.83
Tinetti cut-off (<21p)	*n* = 2 (17%)	0%	1.0
Neuropathy deficit score (NDS)	1.5 ± 1.2p	0.17 ± 0.7p	0.73
NDS cut-off (>3p)	*n* = 2 (17%)	1 (8.5%)	0.5
HADS-A	3.67 ± 2.9p	1.6 ± 1.3p	0.28
HADS-D	3.33 ± 3.3p	1.33 ± 0.55p	0.21
FESI (16–64p)	19.25 ± 3.8p	0.75 ± 0p	0.16
PASE (0–793p)	97.5 ± 43.4p	1.5 ± 16.1p	0.48
Chair-rising test (CRT)	10.83 ± 2.5s	1.25 ± 0.4s	0.057
Timed up and Go (TUG)	8.3 ± 2s	0.84 ± 0.8s	0.21

p: points; s: seconds.

#### 3.1.4 Longitudinal analysis of risk factor changes over 24 weeks (completers; *n* = 8)

Changes in the intraindividual risk factor profile of the eight participants who completed the study were assessed descriptively. In this subsample, no falls occurred during the observation period of 6 months. The mean cumulative oxaliplatin dosage was 1,013 ± 36 mg/m^2^ at T4.

None of these participants developed clinically manifesting polyneuropathy (mean NDS score: 1.25 ± 1.5p; cut-off: 3), with only marginal progress by the end of the study (ΔNDS T1–T4: +0.25p; *p* = 0.57). Functional assessments of mobility, balance, and strength remained stable between T1 and T4 (Tinetti: T1: 25.75 ± 2.96p; T4: 26.9 ± 2.23p; *p* = 0.22; cut-off: <21) or even slightly improved (stand-up test: T1–T4: 11.9 ± 1.9 s vs 9.9 ± 1.5 s; *p* = 0.046) at the end of the study. The screening for anxiety and depression (HADS) remained below the threshold (7 P) until the end of the study. Concerns about falling (FES-I) remained almost unchanged at about 17 points near the minimum level (16p).

Self-assessment of weekly activity (PASE) improved from T1 to T4 (PASE T1–T4: 106.8 ± 55p vs 158.5 ± 57.2p; *p* = 0.018) and reached the reference range of a healthy age-matched population (= 144p) [60].

## 4 Discussion

This prospective observational study investigated the effects of oxaliplatin-based CIPN on functional fall risk in patients with gastrointestinal tumours over a period of 6 months.

### 4.1 Main results

Participants did not develop clinically manifest polyneuropathy during the early phase of oxaliplatin-based chemotherapy (time interval, 0–3 months) and falls occurred exclusively in participants with high pre-existing morbidity indicated by a high fall risk index (>4 RF/person) and mild pre-existing polyneuropathy. While physical performance tests failed to predict falls, the PASE-self-assessment provided useful and detailed information about the physical status of outpatients undergoing chemotherapy. Polypharmacy, anxiety (HADS), and fear of falling (FES-I) were more frequent at baseline in participants who discontinued the study.

### 4.2 Impact of peripheral neuropathy on falls

Independent of the aetiology of polyneuropathy, polyneuropathic sensorimotor deficits impair balance and are a well-recognised risk factor for falling [32,33,34,35].

The neurotoxic effects on the peripheral nervous system in cancer therapy are a relevant dose-limiting factor in cancer therapy, with CIPN incidences of 70% depending on the chemotherapeutic agents [10]. The association between CIPN and falls has been documented at various stages of the antineoplastic therapy but the direct functional relationship remains unclear. Previous study data are heterogeneous in respect of the applied chemotherapeutic agents, cancer population, and CIPN assessment (e.g., rating scales) [15,17,22].

In our prospective study, we assessed sensorimotor symptoms by clinical neurological examination and found no relevant progress of the initial neuropathic score (NDS) within the first 3 months of the oxaliplatin-based chemotherapy. Literature on the neurotoxic profile of oxaliplatin predominantly describes sensory neuropathies with dosages exceeding >540 mg/m^2^ [36], while the mean dose in the present study was 562 mg/m^2^. Furthermore, we had to adjust the time interval provided for correlation analysis from 6 to 3 months because of the high dropout rate (60%). A higher oxaliplatin dosage was reached only in those participants that completed the study (*n* = 8) of which only 50% reached oxaliplatin doses above 1,000 mg/m^2^ (max. 1,500 mg/m^2^). High-grade and persistent CIPN with motor dysfunction, including impaired coordination and mobility, is described with advanced oxaliplatin administration above 1,000 mg/m^2^ or 12 cycles [12,13,37,38,39]. The small number of participants combined with the low cumulative doses – only slightly higher than the mean minimum neurotoxic dose – are the most likely reasons that we found no relevant CIPN.

Nevertheless, our clinical neurological screening detected mild sensorimotor deficits at baseline in 40% of participants that were not pre-diagnosed. In this regard, our retrospective analysis confirmed that pre-existing polyneuropathy is a risk factor for future falls. Our prospective neurological evaluation provided a more nuanced view of the aetiology of sensorimotor deficits in the early chemotherapy intervals. CIPN is commonly assessed using subjective rating scales and retrospective examinations, which may be insensitive in distinguishing between prior polyneuropathy as a predictor of falls and the development of CIPN [40]. Thereby, our findings confirm recommendations in oncological treatment that a brief neurological examination before and during ongoing chemotherapy is essential in supportive oncological management [41].

### 4.3 Falls

Falls in oncology are more common than in the general population because cancer and its treatment put oncology patients at risk of severe impairment; in particular, neurotoxic chemotherapy drugs and an advanced stage of cancer are specific risk factors in oncology [15,17]. Clinically, impairment in ADL is strongly associated with falls in older adults with cancer and is a dynamic risk factor during chemotherapy [42,43]. Moreover, the risk of falling in oncology patients increases with the number of comorbidities [1]. Although fall risk is multifactorial and may vary over time, continuous and time-efficient screening has not yet been implemented in outpatient oncology. In this way, the fall risk index applied in our study summarises established markers of morbidity and frailty in hospitalised and outpatient oncology settings such as age, medication type and number, use of assistive devices or visual impairment [7,42,44]. Among community-dwelling older persons, the predicted 1-year risk of falling increased to 80% when four or more risk factors were present [31]. All fallers in our study exceeded the threshold of four risk factors and were identified as high-risk at baseline assessment, whereas none of the included risk index variables were independently associated with falls. Our results therefore underline the multifactorial aetiology of falls in an outpatient oncological setting. The characteristics of falls in our specific cohort did not differ from those in geriatric patients [45].

### 4.4 Measurements of physical performance

In contrast, previous studies proved the Tinetti test as a solid screening and follow-up instrument for mobility and fall risk in populations with severe physical limitations and in geriatric individuals [46,47,48]. Previous evaluations described single-task instruments (e.g., TUG) as well as physical performance batteries as feasible methods of performance evaluation to assess the potential for falls in geriatric oncology clinics [42,49].

In contrast, our clinical performance measurements did not detect changes in functional fall risk. We found high to maximal scores in mobility and balance tests (TUG, Tinetti) and robust scores for lower limb muscle strength (CRT) that remained almost unchanged over 3 and 6 months, indicating no physical deterioration. These results are most likely due to ceiling effects, which are consistent with previous evaluations, e.g., of the Tinetti test, which found low sensitivity in predicting falls in individuals with mild balance disorders due to coarse scaling [48]. Although we accounted for age-related performance thresholds on other tests, such as the TUG tool, there were no significant results related to fall risk [50].

We summarise that the selected geriatric assessment tools are not applicable to our predominantly younger (mean age of cohort: 59 years) and very mobile adults at the start of oxaliplatin treatment. In future studies, mobility assessments using more finely graded scales, such as the Berg Balance Scale [51], may be more appropriate to screen physically fit adults with a cancer diagnosis.

### 4.5 Screening physical activity during oxaliplatin treatment

In the present study, the PASE proved to be a discriminative screening tool for physical functioning during the first 6 months of chemotherapy. PASE has been shown to be an objective, discriminative, and dynamic instrument for physical activity and health-related quality of life in various cancers [52,53,54] and in outpatient oncology [55,56,57].

In line with previous studies, PASE underlined – in contrast to the functional measurements – that cancer patients are physically less active compared to a non-cancer population [54,58]. Since physical activity is directly linked to quality of life [59,60] and tumour survival [61,62,63,64], it should be monitored during active cancer therapy. PASE has been validated for this purpose with objective markers of functional health such as grip strength, balance, and lower limb strength [65]. Even among the more physically fit participants that completed the study, PASE still discriminated changes in leisure time and household activities well; that is, no ceiling effect was detected. High discriminatory power of assessment tools may become even more important in the future, as early cancer diagnosis in combination with increasing safety and more favourable side effect profiles of cancer therapies are expected to result in a higher prevalence of cancer survivors with low functional impairment in long-term clinical supportive management. In contrast, scales rated by clinicians such as the Karnofsky performance status, and ECOG, which remain integral to health assessment in clinical disease management and research, have limited predictive value for chemotherapy-related toxicity in patients with good to excellent health [66,67].

Therefore, we recommend PASE not only for prognostic evaluations, but also as a therapeutic tool, since self-monitoring increases the individual awareness of patients to maintain activity during chemotherapy [68].

### 4.6 Baseline comparison of dropouts versus completers

In the present study, high levels of psychological distress and polypharmacy at baseline were correlated with study discontinuation. In addition to fear of falling and anxiety as major causes of limited physical activity [69], cancer and treatment-related side effects are also barriers to daily physical activity [70]. Targeted interventions can support patients with poor health-related behaviours and attitudes to avoid or reduce the additional risks associated with a new cancer diagnosis [71].

In addition to the above-mentioned psychological factors, participants who dropped out of the study had a higher incidence of polypharmacy than those who completed the study. Polypharmacy is not only a marker of morbidity but also a strong predictor of falls [72,73], as reflected in our index screening for falls. This may be due to the fact that cancer patients are more susceptible to adverse drug interactions and are particularly likely to take unfavourable drug classes or combinations as part of their cytostatic treatment [44].

### 4.7 Limitations

The single-centre approach extended participant recruitment over a two-year period. The elaborate, in-depth examinations in our study were time-consuming and required a high individual effort for participants during chemotherapy as the main barrier to study participation. The small sample size and resulting low statistical power limited our ability to detect potentially weaker associations between the selected objective markers of physical functioning and chemotherapy-induced neurotoxicity. Therefore, results must be interpreted cautiously.

We did not include measures of postural sway, which would have allowed a more accurate assessment of the effects of latent neuropathy (i.e., even in the absence of CIPN) on balance control and indirectly on fall risk [74].

Because of the limited value of oncological scales such as Karnofsky or ECOG [66,67], with respect to our study objectives, we focused on more specific assessment tools, but this may limit the comparability of our study cohort with other oncological study cohorts.

This study was intended as an initial project to determine whether CIPN-associated fall risk could be appropriately assessed using physical performance tools. As we were confronted with the limitations of these tools, the focus of further investigations in an outpatient oncology setting may be to verify and validate the fall risk index as a preventive assessment tool.

## 5 Conclusions

In this prospective observational cohort study, we could show that pre-chemotherapy conditions contributed more to falls than treatment-related factors in outpatients undergoing oxaliplatin-based chemotherapy. Therefore, a fall risk index including general frailty and morbidity markers [31] might be a promising predictor of falls and should be implemented and validated in a larger multicenter trial. Moreover, we recommend self-monitoring of physical activity during chemotherapy (PASE) since self-monitoring increases the individual awareness of patients to maintain activity during chemotherapy [68].
